# Review of Mindfulness-Related Interventions to Modify Eating Behaviors in Adolescents

**DOI:** 10.3390/nu11122917

**Published:** 2019-12-02

**Authors:** Michael Omiwole, Candice Richardson, Paulina Huniewicz, Elizabeth Dettmer, Georgios Paslakis

**Affiliations:** 1 Toronto General Hospital, University Health Network, Toronto, ON M5G 2C4, Canada; s1417571@sms.ed.ac.uk (M.O.); Candice.Richardson@uhnresearch.ca (C.R.); huniewicz.paulina@gmail.com (P.H.); 2 Department of Psychology, The Hospital for Sick Children, Toronto, ON M5G 1X8, Canada; elizabeth.dettmer@sickkids.ca; 3 Department of Peadiatrics, University of Toronto, Toronto, ON M5T 1R8, Canada; 4 Department of Psychiatry, University of Toronto, Toronto, ON M5T 1R8, Canada

**Keywords:** mindfulness, eating disorders, adolescents, DBT, ACT, obesity

## Abstract

There are few well-established treatments for adolescent eating disorders, and for those that do exist, remission rates are reported to be between 30 and 40%. There is a need for the development and implementation of novel treatment approaches. Mindfulness approaches have shown improvements in eating disorder-related psychopathology in adults and have been suggested for adolescents. The present review identifies and summarizes studies that have used mindfulness approaches to modify eating behaviors and to treat eating disorders in adolescents. Focused searches were conducted in Embase, Medline, and PsycINFO, and identified articles were checked for relevance. A small number of studies (*n* = 15) were designated as appropriate for inclusion in the review. These studies were divided into those that focused on the promotion of healthy eating/the prevention of disordered eating (*n* = 5), those that concentrated on targeted prevention among high risk adolescents (*n* = 5), and those that focused on clinical eating disordered adolescents (*n* = 5). Thirteen of the 15 studies reviewed reported at least one positive association between mindfulness treatment techniques and reduced weight/shape concerns, dietary restraint, decreased body mass index (BMI), eating in the absence of hunger (EAH), binge eating, increased willingness to eat novel healthy foods, and reduced eating disorder psychopathology. In summary, incorporating mindfulness to modify eating behaviors in adolescent non-clinical and clinical samples is still in the early stages, with a lack of data showing clear evidence of acceptability and efficacy. Further studies and preferably controlled conditions are warranted.

## 1. Introduction

Nutrition is critical for healthy childhood and adolescent development [1], yet suggested dietary guidelines are rarely met by youths [2]. Non-adherance is associated with a host of negative health effects, and is one of the main factors leading to the increasing prevalence of obesity [3]. Prevalence rates among American children ages 2 to 5 years has increased from 11% in 1999 to 14% in 2016 [3], and more than one third of American adolescents are considered overweight or obese [4]. Poor dietary habits during childhood and adolescence may be carried into adulthood, leading to type 2 diabetes, cardiovascular disease, and reduced quality of life [5]. Furthermore, youth who are overweight or obese are more likely to experience weight-related stigma and body-based bullying by their peers [6]. Weight-related bullying and negative conversations about body-related issues have been found to significantly impact adolescents’ body satisfaction and eating behaviors, and have ultimately been associated with increased risk for the development of eating disorders [7].

Eating disorders (ED) affect a significant portion of the global population, with lifetime prevalence rates for the three major types, anorexia nervosa (AN), bulimia nervosa (BN), and binge eating disorder (BED), collectively ranging from 1.93% based on a recent study using the DSM-V criteria in the United States (US) [8] up to 10% across different studies conducted among European women [9]. Anorexia nervosa (AN) is characterized by restrictive eating patterns, being underweight, and the fear of gaining weight. Patients with AN are capable of suppressing food intake, even in the presence of strong physiological signals of hunger. Bulimia nervosa (BN) is characterized by regular episodes of binge eating with loss of control, followed by compensatory behaviors such as vomiting or laxative use in an effort to control weight. Binge eating and loss of control also occur in binge eating disorder (BED), but are not followed by compensatory behaviors; obesity or overweight are common [10]. The diagnosis of ED in children and adolescents can be particularly challenging, as these age groups often are not able to reflect on cognitions typically associated with these disorders (fear of weight gain, concerns about body image, etc.) and may instead present with unspecific physical symptoms such as nausea, choking on food, or postprandial abdominal pain. The unique presentation of children and adolescents with eating disordered behaviors has been, in part, acknowledged in the DSM-V by the introduction of revised diagnostic categories, e.g., the avoidant/restrictive food intake disorder (ARFID) [10].

Studies have reported that the age of onset for ED has been decreasing [11], with a noticeable increase in children under 12 presenting for treatment, and a reduced female-to-male ratio in young patients with restrictive ED [12,13], thus raising concerns for early treatment [14]. For AN in youth, some evidence supports adolescent focused therapy (AFT) and cognitive behavior therapy enhanced (CBT-e) approaches [15]. However, family therapy with a behavioral focus (family treatment-behavior; FT-B) is the most established treatment for adolescents with AN [16]. Maudsley family therapy [17] is one of the most widely known FT-B interventions. There are no well-established treatments for adolescents with BN, BED, or ARFID [15,16]. FT-B with supportive individual therapy is considered possibly efficacious in the treatment of adolescent BN. For adolescent BED, CBT interventions have shown some efficacy, while investigations into the futility of other treatment approaches (e.g., interpersonal therapy; IPT) for adolescent BED have also been recently published [16,18,19]. Behavioral plans, CBT, and family interventions are implemented clinically to address ARFID, but empirical studies of efficacy are missing. The current approaches lead to remission rates in adolescents with ED of 30–40% [20]. Thus, there is a clear need for the development and implementation of novel treatment approaches.

Mindfulness, traditionally the result of meditation practice, is described as self-awareness emerging by purposefully paying attention to internal and external moment-to-moment experiences—associated with emotions, thoughts, and bodily sensations—without judgement [21]. Mindfulness has played a major part in Eastern philosophies for hundreds of years and has been increasingly popularized in the West to promote wellbeing, both within and outside the realm of mental health. Mindfulness-based approaches, such as mindfulness-based stress reduction (MBSR) [22], mindfulness-based cognitive therapy (MBCT) [23], or mindfulness-based relapse prevention (MBRP) [24], promote mindfulness through formal meditation practice. Mindfulness-informed approaches, such as the dialectic behavioral therapy (DBT) [25] or acceptance and commitment therapy (ACT) [26], apply non-meditation-based techniques to address mindfulness [27]. A recent analysis of RCTs (randomized controlled trials) involving mindfulness-based interventions illustrated how these interventions can improve cognitive (e.g., sustained attention, working memory), affective (e.g., reducing rumination), and interpersonal outcomes, as well as mental and physical health [28]. The evidence supports the use of mindfulness as an intervention to improve chronic pain management, reduce depression relapse rates in at-risk individuals, and facilitate substance abuse outcomes [22,23,28].

With regard to eating behaviors and disordered eating, a great number of individual studies using mindfulness-based and -informed interventions in adult populations, as well as reviews, have been published showing improvements in eating disorder-related psychopathology [29,30]. Preliminary data in women with ED provide evidence for improvements in body image perception, emotional and external eating following MBCT [31]. Mindfulness meditation approaches have also been added to existing ED treatments [32,33,34]. Finally, ED-specific mindfulness-based treatments have been developed. For example, Mindfulness-Based Eating Awareness Training (MB-EAT) was developed to treat BED. It was conceptualized based on the notion that patients with BED engage in eating behaviors that are steered by external non-nutritive stimuli, rather than the physiological signals of hunger and satiety. Thus, the program addresses self-regulation by meditation practices that involve the perception of emotional vs. physiological triggers of appetite and has been shown to reduce binge eating and preoccupation with food [35]. DBT in patients with ED has been applied in a great number of studies [36], although this approach is recommended for patients with BN or BED with rather low to moderate illness severity and the absence of significant comorbidity [37,38]. DBT has, howeverm been proposed as a viable option for the treatment of ED concurrent with substance abuse [39]. ACT has been recognized as an important therapeutic approach to influence eating behaviors in a manner that differs from standard CBT [40].

There are mindfulness-based or -informed approaches to modify eating behaviors in adolescents in the literature; up to now, however, there has been no attempt to summarize and synthesize the current evidence. Thus, the present review aims to identify and summarize various studies of all types of mindfulness as a method of modifying eating behaviors and a method to treat ED in adolescents. A further aim is to compare studies and find evidence to support the feasibility, acceptability, and effectiveness of mindfulness-based and -informed interventions as modifiers of eating behaviors in adolescents, while at the same time looking at the limitations in experimental study designs.

## 2. Materials and Methods

Focused searches were conducted by a health sciences librarian (MA) in Embase, Medline, and PsycINFO using subject headings and keywords for the three concepts: Mindfulness; eating disorders or eating; and adolescents, teenagers, or young adults. Results were limited to human subjects, English language publications, and publication date up to 8 July 2019. A total of 353 database records were downloaded for reviewers to screen for relevance. M.O., C.R., and P.H. independently reviewed all titles and abstracts to identify relevant articles prior to obtaining full text of selected articles. Search strategies can be found as Supplementary Material (Table S1).

## 3. Results

After the removal of duplicates, 236 articles were identified as potential references. Of these, *n* = 169 were excluded for not fulfilling the inclusion criteria; these were mostly studies performed in adult cohorts (*n* = 146). Furthermore, 5 case reports, 5 publications of study designs, 12 reviews, 29 books/book chapters, 7 dissertations, and 1 study in children were excluded. In addition to the search results of the databases, we identified a further seven relevant articles through cross-referencing. In the end, 15 publications were included in the final review; of these, 13 studies described mindfulness-based and -informed interventions and 2 studies assessed and associated dispositional mindfulness with eating behaviors. There were five studies focusing on promotion of healthy eating/prevention of disordered eating, five studies concentrating on targeted prevention among high risk adolescents, and five focusing on clinical (eating disordered) adolescent cohorts. All of the studies included in this work are presented in Table 1.

### 3.1. Studies with Focus on Prevention (Non-Clinical Adolescent Cohorts)

Johnson et al. [41] conducted a randomized control trial to evaluate the effect of the .b (“Dot be”) Mindfulness in Schools curriculum on anxiety, depression, and eating disorder risk factors among middle and high school students. Classes were randomized and students subsequently received eight weekly lessons of either a mindfulness intervention targeting anxiety, depression, weight/shape concerns, and wellbeing (*n* = 132) or standard curricular lessons (*n* = 176). On a Likert scale from 0–10, the average acceptability of the mindfulness intervention (in terms of enjoyment, interest, amount learnt, and likelihood of using the techniques) was 6.5 among students and 9 among teachers. Despite the high acceptability of the program among students and teachers, no significant improvements in anxiety, depression, weight/shape concerns, or wellbeing were found immediately post-intervention or at 3-month follow-up. Anxiety was also found to be higher in the mindfulness group compared to the control group at follow-up for males, and those with low baseline levels of weight/shape concerns or depression [41].

Salmoirago-Blotcher [42] sought to determine the feasibility and acceptability of integrating mindfulness training (MT)—a modification of the MBSR program for adolescents-into school-based health education, and examine its effects on health-related behaviors. Two high schools were randomized to health education and MT or health education and an attention control condition, which focused on topics of wellness, self-esteem, and mental health. Both conditions involved one 45-min session per week for 8 weeks. Information regarding dietary habits and physical activity levels were collected from students who provided consent (*n* = 53) at baseline, post-intervention, and 6-month follow-up. At both post-intervention and follow-up, MT was found to increase physical activity levels, especially among males and adolescents who exhibited higher physical activity levels at baseline. However, there was no significant differences between MT and attention control in terms of dietary behaviors, such as the intake of fruit and vegetables, sodium, and sugar-sweetened beverages [42]. In terms of program acceptability, 77% of students were satisfied with the mindfulness program.

Atkinson and Wade [43] conducted a comparison study on the feasibility, acceptability, and efficacy of a dissonance-based intervention (challenging thin-ideal internalization and teaching body activism) vs. a mindfulness-based intervention in preventing or reducing weight and shape concern, dietary restraint, thin-ideal internalization, and ED symptoms in female adolescents. A total of *n* = 340 adolescent girls aged 14–18 were included in the analysis, all randomly selected from single-sex girl high schools. Participants were divided into three groups: Mindfulness-based intervention (*n* = 135), dissonance-based intervention (*n* = 108), and a control group (*n* = 97). No significant differences between mindfulness-based and dissonance-based interventions on ED psyhopathology were found. However, when interventions were delivered by expert (optimal) facilitators, adolescents who received mindfulness showed significant reductions in weight and shape concern, dietary restraint, thin-ideal internalization, and ED symptoms compared to the control group at 6-month follow-up. Overall, there was only moderate acceptability for either intervention method, especially the mindfulness-based interventions by students and teaching staff [43].

Turner and Hingle [44] evaluated the feasibility, acceptability, and utility of a mindfulness-based mobile app designed to improve awareness of eating behaviors, physical activity, and sleep in adolescents between the ages 14–18 years. Participants (*N* = 15) were prompted to watch mindfulness-based videos within the app once a day, every day for 6 weeks. The videos, ranging from 2 to 15 min, used storytelling to provide techniques to evoke a state of mindfulness, support guided-practice, and encourage incorporation of mindfulness into life as independent practice. The participants were predominately Hispanic (60%), of normal BMI (80%), and male (53%). Outcomes were measured by in-app and post-study surveys, rated 1–5 on a Likert scale. The authors then conducted descriptive statistical analyses. Adolescents reported increased awareness of eating behaviors (4.1); however, only sometimes found themselves adhering to mindful eating with real foods (3.1). The app was positively rated (3.8) by participants and maintained daily usage rates between 55 and 73%, highlighting its feasibility and acceptability [44].

Hendrikson and Rasmussen [45] looked at both adolescents and adults in a study that examined the strength of the relationship between age, obesity, and impulsive choices for food and monetary outcomes. Of 348 participants, 172 were adolescents who were recruited from two local schools, a youth mailing list, Craigslist, and mass email associated with local news. Participants were assessed using the Food Choice Questionnaire (FCQ) and Monetary Choice Questionnaire (MCQ) before and after either a short mindful eating training to determine the impact on delay discounting for food and money choices, or a control condition. As a main finding, the mindful eating training affected food-related decisions (more self-control), but had no effect on decisions related to money [45].

### 3.2. Studies on Targeted Prevention (High Risk Cohorts)

Annameier et al. [46] examined the effects of an individual’s inherent (dispositional) mindfulness on eating behaviors among overweight and obese (BMI ≥85th percentile) adolescent girls (*n* = 107) at risk for type 2 diabetes (with a family history of diabetes). Using two standard laboratory meal tests, the authors examined eating when hungry and eating in the absence of hunger (EAH), which is defined as the intake of palatable food in response to emotional or external cues in the absence of physiological hunger. Each participant was given a buffet lunch meal, consisting of 11,000 calories worth of multiple lunch food items (e.g., sandwiches, fruits, pretzels, and chips) and instructed to eat until no longer hungry. Additionally, to assess EAH, one hour later, participants were presented with an array of highly palatable, novel foods (e.g., popcorn, candy, and ice cream) and instructed to taste and rate their liking of the foods, and eat as much as they would like. While they found an inverse relationship between dispositional mindfulness and calorie intake during the EAH paradigm, there was no significant relationship between mindfulness and energy intake when eating in a state of hunger. Rather, girls who had reported recent loss-of-control eating (LOC eating), defined as perceived overeating accompanied by a subjective sense of not being able to control what is eaten, consumed more calories when hungry, regardless of dispositional mindfulness [46].

Pivarunas et al. [47] conducted a similar study among overweight and obese (BMI >85th age percentile) adolescent girls aged 12–17 years (*n* = 114) at risk of type 2 diabetes in order to examine the relationship between dispositional mindfulness and binge eating, as well as dietary restraint, and eating, shape, and weight conerns. The Relative Reinforcing Value of Food Task was administered to determine the reinforcing value of each participant’s preferred snack food in relation to one of three alternate rewards. Participants were asked to engage in a hypothetical amount of work to obtain either their preferred snack food or an alternate reward. The amount of work required to obtain the food reinforcer increased each round, while the work required for the alternate reward was held constant. The point at which the participant switched from choosing the preferred snack food to the alternate reward is equivalent to the reinforcing value of that food. The authors found that dispositional mindfulness was associated with lower odds of binge eating, as assessed by the EDE-Q (Eating Disorder Examination-Questionnare). Additionally, mindfulness was found to be inversely associated with eating concerns, EAH in response to fatigue or boredom, and higher food reinforcement compared to physical activity [47].

Daly et al. [48] assessed the feasibility and acceptability of a mindfulness eating intervention (MEI) among adolescent girls by comparing BMI and the promotion of mindful eating to a control group at baseline, post-intervention, and 4-week follow-up. All participants (*n* = 37) were Latino females, aged 14–17 years, with a BMI >90th percentile (clinically classified as overweight/obese). Participants were randomly allocated in a 1:2 manner into either the 6-week MEI group focused on satiety cues (*n* = 14) or a normal care control group that was given a single nutrition and physical health education session (*n* = 23). Participant retention in the MEI and control groups was 57% and 65%, respectively; due to loss to follow up, only 23 participants (MEI: *n* = 8, controls: *n* = 15) were included in analysis. However, all MEI group participants attended every session and were asked what would improve program absenteeism at the end of the study. The remaining participants concluded attrition was due to the perceived fear of peer reactions/stigma (*n* = 4) and lack of readiness to change due to the presence of social barriers in this population (*n* = 2). There was a significant reduction in BMI among the MEI participants in comparison to the control group, whose weight even increased [48].

Shomaker et al. [49] conducted a randomized controlled study to assess the feasibility and acceptability of a standardized mindfulness-based intervention known as Learning to BREATHE among adolescents (*n* = 54) at risk for excess weight gain. The curriculum was derived from MBSR and was adapted for adolescents with experiental activities and guided discussions to teach skills. The control condition was a time-matched health education program group (*n* = 25). The authors hypothesized better outcomes in the mindfulness arm (*n* = 29) in promoting an overall healthier lifestyle, improved stress management, executive function, reduced food reward sensitivity, and less weight and fat gain. Eligible participants were between the ages of 12–17, had a BMI ≥70th percentile for their age/sex, had two biological parents with reported obesity, and were free of psychiatric symptoms that would require treatment. While all participants reported that the mindfulness-based program was helpful, there was no significant difference between the mindfulness intervention and health education program in regard to BMI/adiposity outcomes and stress levels. Acceptability ratings, measured on a Likert scale from 1–5, were above average throughout the study, although the health education group had higher acceptability ratings [49].

Kumar et al. [50] conducted a pilot RCT to evaluate the feasibility, acceptability, and efficacy of a family-based mindful eating intervention (MEI) in adolescents with obesity against standard dietary counseling (SDC). Weight and cardiometabolic risk markers were assessed in participants. Participants were between the ages of 14–17 years with a BMI ≥95th percentile for their age and sex. Twenty-two participants and their parents were randomized in a 1:1 ratio into either MEI or SDC groups; MEI was administered in four 90-min sessions over a 10-week period and SDC was provided at baseline, 12 weeks, and 24 weeks. There was an increase in awareness in the MEI group at 24 weeks and a decrease in distraction during eating at 12 weeks; this was not found in the SDC group. There were no dropouts in the MEI group. Interestingly, results showed that high density lipoprotein (HDL) cholesterol was increased in the SDC group in comparison to the MEI group. Thus, family-based MEI showed feasibility and acceptability in adolescents with obesity and their parents, as shown by the lack of dropouts, despite the necessity for more frequent visits than in the SDC group [50].

### 3.3. Studies in Clinical (Eating Disordered) Adolescent Cohorts

Mazzeo et al. [51] assessed satisfaction, feasibility, and preliminary outcomes of a standardized LOC eating intervention in ethnically diverse adolescent girls by comparing a DBT intervention called Linking Individuals Being Emotionally Real (LIBER8) against a weight management control group (2Bfit). Of the 45 participants, all were girls (44.4% white, 42.2% black), aged 13–17 years, who met criteria for LOC eating or BED in children. Participants were assessed on eating behaviors after each meeting, upon completion of intervention, and at a 3-month follow-up. The authors found that LIBER8 and 2Bfit yielded comparable results, both producing significant reductions in ED-related thoughts (eating concern, shape concern, dietary restrictions); however, LIBER8 showed a higher feasibility and satisfaction rate amongst participants [51].

Fischer and Peterson [52] conducted a pilot study in order to examine the effectiveness of an outpatient DBT program for adolescents girls with BN symptoms and non-suicidal self-injury (NSSI) or past suicide attempts. Ten adolescents were enrolled in the study, and seven completed the full 6 months of treatment. All participants were provided with outpatient DBT, including weekly: Skills training group, meetings with a therapist, and consultation team meetings, as well as access to telephone coaching between sessions. Participants reported significant reductions in ED symptoms (as measured by EDE scores), self-harm, and frequency of both binge eating and purging episodes; these reductions were maintained at 6-month follow-up. Additionally, at 6-month follow-up, three of the seven treatment completers no longer met criteria for an ED [52].

Pennell et al. [53] published preliminary outcomes of integrating a DBT-informed program with family-based treatment for adolescents with eating disorders (*n* = 24) treated within a day hospital program. In addition to existing FBT, participants attended DBT-informed therapy groups that focused on: Distress tolerance, mindfulness, emotion regulation, and interpersonal skills. Participants were found to have increased weight and percentage of ideal body weight, as well as reduced binge–purge behaviors at discharge. Additionally, readmission rates over a 2-year period were found to be low (25%), and of those readmitted (*n* = 6), only two had finished the DBT program [53].

Similarly, Peterson et al. [54] examined the efficacy of a DBT skills group as an adjunct to FBT among adolescents girls (*n* = 18) with restrictive EDs. In conjunction with FBT, participants completed weekly DBT skills group based on the 24-week Standard Adults DBT Skills Training Schedule, which included six sessions of mindfulness over the 6-month period. Overall, they found increases in adaptive skills (emotion regulation and distress tolerance) and percent expected body weight, as well as decreases in dysfunctional coping strategies, binge eating, and ED symptoms (measured by EDE Q-scores). The authors concluded that overall, an adjunct DBT skills group seems feasible based on attendance rates (12/18 attended the majority of sessions); notably, of the six participants that dropped out, only two cited program dissatisfaction as the reason (three required higher levels of care, one had transportation issues) [54].

Finally, in a pilot study, Johnston et al. [55] assessed an intensive outpatient (IOP) treatment model combining both Maudsley-based family therapy and group DBT skills training among female adolescents between the ages of 12–17 suffering from ED, by measuring the physical and psychological status of participants upon admission, discharge, and at 3-month follow-up. Participants (*n* = 55) attended an adolescent IOP ED program three times a week for 3–4 h, with each family attending the program for 7–8 weeks, once a week for 45 min. The program focused on DBT skills from the mindfulness and emotion regulation modules, as well as distress tolerance and interpersonal effectiveness. Participants who completed the program showed a significant increase in weight and significant decrease in ED psychopathology, while at a 1-year follow-up, 64% of participants were restored to a healthy weight with normal menstruation and sustained decreases in ED psychopathology [55].

## 4. Discussion

We performed a review of published empirical studies focusing on either the prevention or treatment of eating-related behaviors in adolescents using all types of mindfulness interventions. A small number (*n* = 15) of studies were identified as relevant and included in the present paper.

Mindfulness has been defined as the non-judgemental self-awareness that is traditionally achieved by means of meditation [21]. A broader definition of mindfulness treatments includes non-meditative approaches to regulate emotions, as well as strengthen distress tolerance and compassion for oneself and others. Such non-mediative mindfulness interventions have been found to positively impact the outcomes in eating disorder treatments [56]. Adults with high dispositional (“trait”) mindfulness appear to display lower levels of ED symptoms compared to those with lower dispositional mindfulness [57]. While most published studies so far have included adult populations [29,35,58,59,60], there is only a handful of studies providing empirical evidence of effectiveness for mindfulness interventions to modify eating behaviors in adolescents.

We found 15 studies examining the effects of mindfulness-based interventions on modifying eating behaviors in adolescents. Five studies were related to prevention and were conducted using non-clinical (non-eating disordered) adolescent cohorts. Five studies were conducted among high risk cohorts of overweight and obese adolescents [46,47,48,49,50], and two were conducted among girls with LOC eating or binge eating [51,52]. Additionally, three studies were conducted among adolescents with restrictive eating disorders [53,54,55]. Eight studies used female-only samples, while the others consisted of mixed-gendered cohorts. Although the studies’ methodologies were heterogeneous, eight studies [41,42,43,45,48,49,50,51] compared a mindfulness intervention to type of control condition such as an education class. One study compared mindfulness to both a dissonance-based and a control condition [43], and another compared a DBT intervention with a weight management program [51]. Three of the studies used specific mindful exercises targeting eating behavior [45,48,50]. Three studies utilized standard laboratory tasks to measure eating behaviors, while one study used a mindfulness-based mobile app as an intervention [44]. Furthermore, four studies utilized DBT-based interventions [52,53,54,55].

Overall, 13 of the 15 studies reported mild positive effects of mindfulness [43,44,45,46,47,48,50,51,52,53,54,55,61,62]. Two of these studies [43,51] found mindfulness was associated with reduced weight/shape concerns and dietary restraint among adolescent girls, while others found it to be associated with decreased BMI [48], EAH [46], and binge eating [47]. Mindfulness-based DBT interventions were also found to reduce ED psychopathology in four studies conducted among clinical cohorts of adolesents with eating disorders [52,53,54,55].

Ten of the studies included in this review reported on the acceptability of the mindfulness-related interventions [41,42,43,44,48,49,50,51,52,54]. While most of the interventions were reported to be moderately acceptable, overall acceptability was quite low. For instance, Johnson et al. [41] found that while teachers rated the acceptability of the mindfulness program as 9/10 on average, students rated it as 6.5/10. Similarly, Salmoirago-Blotcher et al. [42] found that 77% of students were satisfied with the MT (mindfulness treatment) intervention compared to over 90% among the control intervention of health education, and Shomaker et al. [49] found greater acceptability for the health education (control) program compared to the BREATHE intervention. Additionally, Daly et al. [48] reported retention to be 57% among the MEI condition compared to 65% among controls. Retention rates among clinical cohorts were approximately 70% [52,54]. These results do raise an issue about the way interventions of this kind are delivered to children and adolescents, which may not be adequate. This may call for an improvement in the method and content of delivery of such interventions for this group of recipients [43]—at least as stand alone interventions.

Unfortunately, the limited number of overall studies and the heterogeneity in methodologies and outcomes that do not allow for comparisons and conclusions regarding efficacy. Although four of the studies conducted among clinical cohorts found mild positive effects of mindfulness, none of these studies included a true control group; thus, superiority of the mindfulness-related interventions cannot be proven. Moreover, the one clinical study that did utilize a control condition found reduced eating pathology in both the control and DBT group [51].

Pluhar et al. [63] proposed several points of improvement of mindfulness interventions, such as providing manualized workbooks for participants as after-session homework to continue developing their skills. The use of online technology as a medium to share the in-session exercises and materials could also improve the dissemination of such interventions among adolescents who engage in the use of new technologies, and could increase accessibility while eliminating class absence [63]. The study performed by Atkinson and Wade [43] signifies the importance of skilled experts in the dissemination of these mindfulness-related interventions to produce significant results in effectively modifying eating behavior and reducing symptoms of ED. This finding clearly demonstrates that the delivery of mindfulness-related interventions requires skills, thus making the implementation of education and training programs to foster competent clinicians a necessity. As well, a great deal of work is needed to better understand how to select and tailor specific mindful approaches for different adolescent populations based on developmental stage, learning abilities, and type of disordered eating. For example, a subset of adolescents who struggle with obesity also struggle with ADHD and/or learning disabilities [64]. This may greatly impact their willingness and ability to implement specific mindfulness strategies if not correctly tailored. It is also possible that while a specific mindfulness approach may be helpful for one population of adolescents, it may be counterproductive for another; an adolescent that is experiencing a high level of medication-induced hunger or hyperphagia would not likely benefit from trying to be more mindful of the bodily sensation of hunger, but instead should be mindful when eating.

In regards to hunger, Annameier et al. [46] found no relationship between calorie intake and mindfulness when participants were eating in a state of hunger. Rather, participants’ recent experience of LOC eating seemed to mediate the relationship with intake, as those who reported recent LOC eating consumed more calories when hungry, regardless of dispositional mindfulness. This finding conflicts with Pivarunas et al. [47], who found that deficits in mindfulness were linked to the presence of binge eating. However, the two studies both found an inverse association between mindfulness and eating in the absence of hunger (EAH). Thus, the effectiveness of mindfulness interventions in a state of hunger, a common trigger for LOC and binge episodes, remains unclear and warrants further investigation. Furthermore, the chosen study populations were quite specific: Both studies focused on the effects of mindfulness on the disinhibited eating habits of adolescents who were prone to weight gain and type 2 diabetes. The specific nature of the sample may limit the scope of the study’s application across types of disordered eating or eating disorders.

Interestingly, Salmoirago et al. [42] found that the mindfulness training intervention increased physical activity levels, especially among males, while Johnson et al. [41] found that anxiety levels were higher among males in the mindfulness group post-intervention. These findings, rather surprising at first sight, may allude to the potential for differential effects of mindfulness across genders. Although this may hold important implications, data among adolescent males is sparse; only about half of the studies included in this review included male participants. Additionally, although Daly et al. found that the MEI (mindfulness eating intervention) group had significantly lower BMI post-intervention, the control group had an unexpected increase in weight. While this finding is unusual, the authors point to natural pubescent growth as a possible causal factor [48].

Mindfulness is increasingly being incorporated into evidence-based treatments by therapists treating adolescents with ED [55,65]. Adolescents receiving Maudsley treatment plus DBT skills training showed significantly higher weight gain and a more pronounced decrease in ED symptoms than adolescents in Maudsley treatment alone, even at the 1-year follow-up [55]. Several relevant factors contributing to the effectiveness of mindfulness have been identified including: “A sense of control and choice”, “relating differently to thoughts and feelings”, “feelings towards the self”, and “taking control through understanding, awareness, and acceptance” [66,67]. Mindfulness is proposed to have significant effects on ED psychopathology through managing stress, reducing overeating caused by secondary symptoms such as depression and anxiety, and mitigating food reward sensitivity via systematic mindful exercises such as meditating, mindful breathing, and stability [46]. Futhermore, focusing on mindfulness practices appears to have an impact on brain structure and function, as shown in an increasing number of neuroimaging studies [68,69,70,71].

Studies applying mindfulness interventions in children are scarce. In a recent study, Hong et al. [72] determined the effects of a mindfulness-based intervention on eating enjoyment and behaviors in children (*n* = 65) between 3 and 10 years of age. The intervention showed promising results for mindfulness as a way to diversify and encourage healthier eating habits in young children. However, there might have been a group mimicry mentality, as the children may have felt compelled to follow the lead of the others in the mindfulness condition. Nonetheless, the results also showed that the increased willingness to try healthier food options did not increase the enjoyment about these foods or improve children’s preferences in the long-term [72]. Also recently, the initial validation of the Mindful Eatng Questionnaire adapted for Children (MEQ-C) was published [73]. Further studies in children are warranted.

There were also several limitations among the articles included in the present review. Almost half of the studies in this review included only adolescent females, and of those that included males, they usually comprised less than half of the sample. It therefore remains unclear as to what extent the results can be applied across genders. Additionally, while each study explored the effects of mindfulness on eating behaviors, there was a lack of consistency across studies in terms of specific outcome measures that were assessed (e.g., dietary restraint vs. weight gain) and the methods used to evaluate these behaviors (e.g., self-report vs. laboratory eating task). The heterogeneity in methodologies and outcomes makes it difficult to collate data and formulate a coherent and concise intervention development method that will be effective in changing eating behaviors among adolescents. While sample sizes were generally moderate, some were quite small (e.g., *n* = 18 in [54]) in relation to others (e.g., *n* = 1820 in [62]). Similarly, due to the particular geographic location of their study, Daly et al. [48] only included Latino adolescent girls, which may limit the applicability of their findings to the general population. Additionally, while mindfulness showed some efficacy among non-clinical adolescent cohorts, only one study (i.e., [51]) examined the effects of mindfulness among those with LOC or binge eating. Furthermore, among the studies that focused on high-risk cohorts, the majority examined the effects of mindfulness on EAH, LOC eating, and reducing BMI rather than eating psychopathology on the opposite side of the spectrum (e.g., maladaptive restrictive eating, pronounced weight loss due to malnourishment). While we did find four studies (i.e., [52,53,54,55]) conducted among adolescents with restrictive eating disorders, the interventions were DBT programs that incorporated mindfulness as a skill or component, rather than stand-alone mindfulness only interventions. Similarly, in three (i.e., [53,54,55]) of the four studies, DBT was used in addition to FBT and/or day hospital eating disorder treatment. These treatment modalities include a number of different therapeutic components, which limits the ability to draw conclusions regarding the efficacy of mindfulness alone. As described by Pennell et al. [53], future research is needed to determine which therapeutic components and combinations are most effective in improving patient outcomes. Finally, although we conducted a thorough search for relevant articles in three databases, screening a total of *n* = 353 articles, and also searched for cross-referenced studies, this search cannot be considered exhaustive; thus, single relevant publications may have been missed. In summary, incorporating mindfulness to modify eating behaviors in adolescent non-clinical and clinical samples is still in the early stages with a lack of data showing clear evidence of acceptability and efficacy. There is singular evidence for the efficacy of mindfulness as a preventive initiative in girls and when delivered by professionals. Further studies and preferably controlled conditions are warranted.

## 5. Conclusions

The present review article summarizes several pieces of literature regarding the feasibility, acceptability, and effectiveness of mindfulness-based and -informed interventions for eating behaviors in adolescents. Based on the current evidence, effectiveness for such methods may be expected, but this may only be considered a preliminary assumption based on the very limited currently available evidence. There is still considerable work to be done on increasing the use and acceptability of these interventions to successfully modify eating behaviors in adolescents. Future research is warranted and should incorporate longer and more frequent follow-ups to determine the long-term effects of mindfulness-related interventions on the eating behavior of children and adolescents. A great deal of work is needed to better understand how to select and tailor specific mindful approaches for different adolescent populations. Additionally, factors of parent/caregiver involvement need to be more fully investigated. A parent’s ability and willingness to support the practice of mindfulness strategies in the home are likely essential for short- and long-term use of learned mindfulness techniques.

## Figures and Tables

**Table 1:** Summary of all studies on mindfulness-related interventions to modify eating behaviors in adolescents that were included in the present review. ED: Eating disorder; RT: Randomized Trial, RCT: Randomized Control Trial; AN: Anorexia nervosa; BN: Bulimia nervosa; BED: Binge eating disorder; OSFED: Other specified feeding and eating disorders; ARFID: Avoidant restrictive food intake disorder; EAH: Eating in the absence of hunger; T2D: Type 2 diabetes; HDL: High density lipoprotein; LOC: Loss-of-control; BMI: Body mass index; MT: Mindfulness training; MEI: Mindfulness eating intervention; SDC: Standard dietary counseling; FBT: Family-Based Treatment; DBT: Dialectic behavioral therapy. For details please refer to main text.

Article	Study Design	Sample	*N*	Intervention	Results	Acceptability
**Prevention (Non-Clinical Cohorts)**						
Johnson et al. [41]	RT	High school students	308	.b Mindfulness in schools curriculum (8 weekly lessons) vs. standard curricular lessons	Higher anxiety was found among mindfulness group at follow-up, especially for males and those with low baseline weight/shape concerns or depression	Average acceptability rating was 6.5/10 for students and 9/10 for teachers
Salmoirago-Blotcher et al. [42]	RCT	High school students	53	Health education plus mindfulness training (45 min session/week for 8 weeks) vs. health education plus attention control	MT found to increase physical activity, especially among males and those with higher physical activity at baseline No difference in dietary behaviors	Satisfaction was 77% among MT compared to >90% among attention control
Atkinson and Wade [43]	RCT	High school girls	347	Mindfulness intervention vs. dissonance-based intervention vs. control (classes)	No difference between mindfulness and dissonance conditions Reduced weight/shape concern, dietary restraint, thin-ideal internalization, ED symptoms among mindfulness group compared to control when intervention delivered by expert facilitators	Only moderate acceptability by students and teachers
Turner and Hingle [44]	Single-arm pilot study	Adolescents	15	Mindfulness-based mobile app (videos once/day for 6 weeks)	Reported increased awareness of eating behaviors (4.1/5) and moderate adherence to mindful eating with real foods (3.1/5)	App was rated 3.8/5 Daily usage ranged from 55–73%
Hendrikson and Rasmussen [45]	Pilot RCT	Adolescents	172	Mindful eating vs. DVD control vs. standard control	Mindful eating training affected food-related decisions (more self-control), but had no effect on decisions related to money	N/A
**Targeted Prevention (High Risk Cohorts)**						
Annameier et al. [46]	Cohort study	Overweight and obese (BMI ≥85th percentile) adolescent girls at risk of T2D	107	Standard laboratory eating tests: Eating while hungry and EAH	Inverse association between dispositional mindfulness and energy intake during EAH but no relationship when hungry Previous LOC eating was associated with increased intake when hungry, regardless of mindfulness	N/A
Pivarunas et al. [47]	Cohort study	Overweight and obese (BMI >85th age percentile) adolescent girls at risk of T2D	114	Relative Reinforcing Value of Food Task	Dispositional mindfulness was associated with lower odds of binge eating, and inversely associated with eating concerns, EAH when bored, and higher food reinforcement	N/A
Daly et al. [48]	Two group repeated measures	Latino females, aged 14–17, with a BMI >90th percentile	37	Mindfulness eating intervention (6 weeks, focus on satiety cues) vs. control (single nutrition and physical health education session	Reduction in BMI among mindfulness group compared to control	57% retention among the mindfulness group vs. 65% among controls
Shomaker et al. [49]	RCT	Adolescents aged 12–17 at risk of excess weight gain (BMI ≥70th or parental obesity)	54	Learning to BREATHE vs. health education program	No significant difference in BMI between BREATHE and health education group	Health education intervention had higher acceptability, although both were considered acceptable
Kumar et al. [50]	Pilot RCT	Adolescents aged 14–17 with BMI ≥ the 95th	22	Family-based mindful eating intervention vs. standard dietary counseling	Increased awareness and decreased distraction while eating among the MEI group Increase in HDL cholesterol among SDC group	No dropouts
**Clinical (Eating Disordered Cohorts)**						
Mazzeo et al. [51]	RCT	Adolescent girls aged 13–17 with LOC eating or BED	45	DBT (LIBER8) vs. weight management control (2Bfit)	Both produced reductions in eating and shape concern and dietary restraint	Higher satisfaction and feasibility among LIBER8 group
Fischer and Peterson [52]	Pilot study	Caucasian adolescent girls aged 14–7 with binge eating (pat month), weight and height within/above normal limits for age, and a suicide attempt or self-injury within past 12 months	10	Outpatient DBT	Significant reductions in ED symptoms, self, harm, and frequency of both binge eating and purging episodes	70% retention rate
Pennell et al. [53]	Retrospective analysis of hospital records	Adolescents aged 13–17 with AN, OSFED, ARFID, or BN	24	6-week DBT-informed program integrated with FBT within an adolescent day hospital eating disorder treatment program	Increased weight and percentage of ideal body weight, as well as reduced binge–purge status at discharge compared to admission Low readmission rate (25%)	N/A
Peterson et al. [54]	Pilot study	Adolescent girls aged 13–18 with restrictive EDs (AN, atypical AN, OSFED)	18	6-month, weekly DBT skills group in addition to concurrent FBT	Increases in adaptive skills (emotion regulation, distress tolerance) and percent expected body weight Decreases in dysfunctional coping strategies, binge eating, and ED symptoms	66% retention rate
Johnston et al. [55]	Pilot study	Adolescent females aged 12–17 with EDs	55	FBT with DBT skills training	Significant increase in weight and decrease in ED psychopathology 64% restored to healthy weight at 1-year follow-up	N/A

## Supplementary Materials

The following are available online at https://www.mdpi.com/2072-6643/11/12/2917/s1: Table S1: Search strategies.
