# Supplementary material for: Review of Mindfulness-Related Interventions to Modify Eating Behaviors in Adolescents
Source: Nutrients. 2019 Dec 2;11(12):2917. doi: 10.3390/nu11122917 (PMC6950168; doi:10.3390/nu11122917)
Supplement: Supplementary file 1 [file nutrients-11-02917-s001.pdf]

Supplementary material:

Table S1:search strategies

Summary
Boolean:
Mindfulness
AND (Eating OR Eating disorders)
AND (Adolescents OR teenagers OR young adults)
Limits: English language publications and Human subjects
Databases: Medline, PsycINFO, Embase
Searches run in July 2019

Search Key

/	At the end of a term a / indicates that the term is a subject heading, to be searched in the subject heading field
*	At the end of a term a * indicates that the database should look for any ending: mindful, mindfulness, mindfully
.mp.	At the end of a term a .mp. indicates that the database should look for the term in the title, abstract, author assigned keywords, subject headings, and a few other fields
exp	At the beginning of a subject heading, an exp indicates that the database should look for this subject heading OR any subject heading below it in the hierarchy: eating behaviour or binge eating or food refusal or dietary restraint or …

Ovid MEDLINE and Epub Ahead of Print, In-Process & Other Non-Indexed Citations <1946 to July 05, 2019>

1 Mindfulness/
2 mindful*.mp.
3 1 or 2
4 Eating/
5 Feeding Behavior/
6 eating.mp.
7 4 or 5 or 6
8 3 and 7
9 limit 8 to "adolescent (13 to 18 years)"
10 Adolescent/
11 (adolescen* or teen* or "young adult*").mp.
12 10 or 11
13 8 and 12
14 9 or 13
15 limit 14 to (english language and humans)

Ovid PsycINFO <1806 to July Week 1 2019>

Search Strategy:

1 Mindfulness/
2 mindfulness-based interventions/
3 mindful*.mp.
4 1 or 2 or 3

5 exp eating behavior/
6 eating.mp.
7 5 or 6
8 4 and 7
9 (adolescen* or teen* or "young adult*").mp.
10 8 and 9
11 limit 8 to 200 adolescence <age 13 to 17 yrs>
12 10 or 11
13 limit 12 to english language

Ovid Embase <1974 to 2019 July 05>

Search Strategy:

1 Mindfulness/
2 mindful*.mp.
3 1 or 2
4 eating/
5 feeding behavior/
6 eating habit/
7 eating.mp.
8 4 or 5 or 6 or 7
9 exp adolescent/
10 (adolescen* or teen* or "young adult*").mp.
11 9 or 10
12 3 and 8 and 11
13 limit 12 to (human and english language)
